# The Functional Consequences of Eukaryotic Topoisomerase 1 Interaction with G-Quadruplex DNA

**DOI:** 10.3390/genes11020193

**Published:** 2020-02-12

**Authors:** Alexandra Berroyer, Nayun Kim

**Affiliations:** 1 Department of Microbiology and Molecular Genetics, University of Texas Health Science Center at Houston, Houston, TX 77030, USA; Alexandra.Berroyer@uth.tmc.edu; 2 MD Anderson Cancer Center UT Health Graduate School of Biomedical Sciences, Houston, TX 77030, USA

**Keywords:** topoisomerase I, G-quadruplex, genome instability

## Abstract

Topoisomerase I in eukaryotic cells is an important regulator of DNA topology. Its catalytic function is to remove positive or negative superhelical tension by binding to duplex DNA, creating a reversible single-strand break, and finally religating the broken strand. Proper maintenance of DNA topological homeostasis, in turn, is critically important in the regulation of replication, transcription, DNA repair, and other processes of DNA metabolism. One of the cellular processes regulated by the DNA topology and thus by Topoisomerase I is the formation of non-canonical DNA structures. Non-canonical or non-B DNA structures, including the four-stranded G-quadruplex or G4 DNA, are potentially pathological in that they interfere with replication or transcription, forming hotspots of genome instability. In this review, we first describe the role of Topoisomerase I in reducing the formation of non-canonical nucleic acid structures in the genome. We further discuss the interesting recent discovery that Top1 and Top1 mutants bind to G4 DNA structures in vivo and in vitro and speculate on the possible consequences of these interactions.

## 1. Introduction

Eukaryotic Topoisomerase I (Top1) is a type IB topoisomerase that binds to duplex DNA (dsDNA) and cleaves a single strand initially forming a phospho-tyrosyl bond at the 3′ end of the cleaved DNA. Self-catalyzed ligation to the 5′ hydroxyl restores the intact double-stranded DNA. Helical torsion is removed by the swivel of the DNA strands between the cleavage and ligation steps. The function of Top1 has recently expanded beyond maintaining torsional homeostasis in the genome. In 1997, Sekiguchi and Shuman reported on the ribonuclease activity of vaccinia and human Top1 and suggested the potential role of Top1 in processing RNA [1]. Later, the endoribonuclease activity of eukaryotic Top1 was shown to be required in its role of removing ribonucleotides incorporated into DNA [2,3,4]. In absence of a functional RNase H2 complex, which initiates error-free ribonucleotide excision repair, Top1-mediated cleavage at single ribonucleotides embedded in a DNA strand leads to mutations and genome instability [5,6]. The role of Top1 in transcriptional regulation was also recently highlighted. Along with the type II topoisomerase Top2, Top1 functions to recruit RNA polymerase to the promoters of highly transcribed genes [7] and is required to maintain the superhelicity of certain inducible promoters [8]. More recently, the role of Top1 as a component of the RNA polymerase complex and as a positive regulator of promoter escape and transcription elongation was described [9]. Here, we review another seminal function of Top1 in reducing the formation of genotoxic DNA secondary structures. For G-quadruplex (G4) DNA, which is a DNA secondary structure formed at guanine-rich sequences, its significance in elevating genome instability in eukaryotic cells and the characterization of DNA helicases relevant in counteracting G4-induced instability have been highlighted by multiple publications. In addition, the emerging evidence implicates G4 DNA in cancer-associated genome rearrangements. Several excellent reviews comprehensively describe these important findings regarding G4 DNA and genome instability [10,11,12]. In this review, we place a particular focus on the interaction between Top1 and G4 DNA structure. Furthermore, we discuss the recent findings indicating that Top1 is a high-affinity G4 DNA-binding protein and speculate on the functional outcome of such an interaction.

## 2. Top1 Binding of Duplex DNA

The first step in the catalytic cycle of topoisomerase reaction is binding to DNA. X-ray structure of human Top1 bound to a DNA duplex shows that two lobes clamp around the B-form double stranded DNA throughout the catalytic cycle [13,14]. Core domains I and II form the “cap” lobe while the core domains III, C-terminal domain, and the linker domain form the “catalytic” lobe. Although a 22-bp duplex DNA was used for the structure determination, Top1 contacts only the central 10 bp of the DNA from the position −4 to +6 (with the cleavage between the −1 and +1 positions). The essential catalytic tyrosine residue (Y723) is covalently attached to the phosphodiester bond 3′ of the −1 position nucleotide. When this residue is mutated to the non-catalytic form phenylalanine, the aromatic ring is closely positioned facing the phosphodiester bond between the −1 and +1 nucleotides. In total, 24 to 26 amino acids make direct contact with the DNA molecule, but there is only one base-specific contact indicating that the Top1–DNA interaction is not strongly sequence-dependent. Further experiments showed that, rather than sequence, superhelicity of DNA determines the strength of Top1–DNA interaction [15]. Using the catalytically inactive mutant with phenylalanine at amino acid 723, it was shown that human Top1 binds preferentially to the superhelical DNA and particularly to the nodes created by the crossing of the two helical dsDNA. In this experiment, Top1 Y723F initially bound to relaxed DNA molecule redistributed to bind the added supercoiled DNA indicating Top1–DNA interaction is dynamic and transient for relaxed DNA. No preference for positively or negatively supercoiled DNA was found. Although the core, the linker and the C-terminal domains participate in DNA binding, a truncated form that contains only the linker and the core domain retained the preference for binding supercoiled DNA. The linker domain was later determined to be important in the preferential binding to supercoiled DNA [16]. Vaccina Top1 is an example of a topoisomerase with a strict sequence requirement for binding; it binds and cleaves at 5′-CCCTT-3′ [17]. For eukaryotic Topoisomerases, however, the sequence requirement is less restricted. Inferring the sites of Top1 binding preference from the sites of Top1 cleavage preference, the consensus for strong cleavage for rat and wheat germ Top1 is 5′ A/T G/C T/A T (from the −4 to −1 position). However, a significant level of cleavage occurs when the −1 position is a C residue [18]. When the Top1 cleavage sites are analyzed by trapping the cleavage complex with the Top1 poison camptothecin (CPT), the sites of strong preference are clearly shifted from its preferential cleavage site in the absence of the intercalating drug [19]. CPT-intercalation into the DNA-Top1 complex requires T at the −1 position and prefers G at the +1 position. 

## 3. The Role of Top1 in Preventing the Formation of G4 DNA and Other DNA Secondary Structures

Non-canonical DNA structures can disrupt processes such as replication, transcription, and DNA repair. An R-loop or the long extensive hybrid between RNA and DNA strands is one such non-canonical nucleic acid structures that is a major source of endogenous genome instability (reviewed in [20]). Top1 function counteracts the formation of extensive R-loops at highly transcribed regions. At the ribosomal DNA repeat loci in the yeast genome, the loss of Top1 leads to disruption of transcription and accumulation of R-loops, both of which are severely exacerbated in absence of the RNA:DNA hybrid processing nucleases RNase H1 and H2 [21,22]. R-loop accumulation in the absence of Top1 function and the elevated genome instability as a consequence were also observed in mammalian cells [23]. 

Apart from the RNA–DNA hybrid or R-loops, DNA can assume structures other than the Watson-Crick double helical, B DNA structure (reviewed in [24]). Duplex DNA, under the condition of increasing supercoils, particularly negative torsional stress, undergoes structural transformation to partially single-stranded DNA [25]. The exposed bases in this context can lead to intrastrand interactions promoting secondary structure formation. For example, base-pairing within a strand of DNA can occur at inverted repeats, forming a basis for a hairpin structure or a cruciform DNA. CAG trinucleotide repeat-containing DNA strands are known to assume stable hairpin structures through the intrastrand base pairing of Cs and Gs. Other unusual, non-B structures include the triplex or H-DNA formed at purine-rich sequences such as GAA trinucleotide repeat loci. Removal of negative torsion by Top1 prevents stretches of single-stranded DNA folding into non-canonical secondary structures such as hairpins. The hairpin-forming CAG trinucleotide repeats are found at human genes associated with the neurological disorders Hungtington’s disease and spinocerebella ataxia. The manifestation of these diseases is dependent on the expansion of the repeat sequences, which in turn is associated with the topological and structural changes at these genomic loci due to non-B DNA structure formation. In a screen of small molecules, Top1-inhibitors were identified as inducing the expansion of CAG trinucleotide repeats in a human cell culture system [26]. This was confirmed by separate work which showed that the knock-down of Top1 significantly elevated the instability of a large CAG-repeat in a human fibrocarcinoma cell line [27]. By removing the supercoils generated during the transcription of these sequences, Top1 likely prevents the formation of pathological non-B DNA structures and helps maintain stability at these unusual repetitive sequences. 

Another non-B structure of interest is the four-stranded G-quadruplex DNA or G4 DNA. Held together by the Hoogsteen bonds among four guanine bases forming a ring-like structure called a G-quartet, G4 DNA is composed of multiple runs of guanines forming multiple G-quartets stacked on top of each other [11]. The significance of G4 DNA as a source of genome instability has recently become evident with the bioinformatics studies finding G4 motifs, along with other non-B DNA forming sequences, to be highly enriched at chromosomal translocation hot spots found in cancers [28,29]. At BCL2 and c-MYC, two genes known to be involved in recurrent blood cancers, G4 motifs are located close to the major chromosomal break point region [28]. A high density of G4 motifs are also present in BCR (B-cell receptor) or the immunoglobulin heavy chain (IgH) loci, which are frequently involved in genome rearrangements and other changes in blood cancers. In mouse B lymphocytes, a decrease in the protein level of Top1 resulted in elevation in the class switch recombination (CSR) at IgH loci [30,31]. Chromosomal translocation between IgH locus and c-Myc is also increased by the knock down of either Top1 or the chromatin remodeler SMARCA4, which is required for the efficient recruitment of Top1 to the chromatin [32]. The decrease in the recruitment of Top1 to the switch regions was concurrent with the increase in the negative helicity at the same locus as measured by the incorporation of modified psoralen molecules that bind preferentially to underwound DNA. CSR initiates with the activation of transcription of the switch sequences that are unusually G/C-rich and contain multiple runs of guanines capable of folding into G4 DNA. Increased propensity for G4 DNA folding at these sequences could explain the elevation in CSR and in IgH/c-Myc translocation observed with reduced Top1 levels. Similarly, when a fragment of IgH switch region was embedded into the yeast genome, the rate of recombination occurring at this sequence was significantly elevated in the absence of Top1 [33,34]. This effect of Top1-disruption on genome instability was G4-specific; the elevation of recombination was observed only when the switch region sequence was placed in the orientation that allows the G-rich DNA strand to fold into G4 DNA. Additional experiments with this IgH switch region-containing recombination reporter system showed that Top1-catalyzed removal of transcription-associated negative supercoils is essential to avert the G4 DNA-induced recombination ensuing upon G4 DNA folding. The importance of Top1 function in preventing the detrimental effect of G4 DNA was confirmed in human cells where Top1 was shown to protect cells from the genotoxic effect of the G4-binding small molecules [35]. 

## 4. Top1 Binding G4 DNA

In addition to playing a role in suppressing the formation of non-B DNA structures by preventing the accumulation of negative supercoils, a handful of studies demonstrated that Top1 can also physically bind to non-B DNA structures including G4 DNA [36,37,38,39]. The first strong evidence of Top1 interaction with G4 structures is described in studies by Arimondo et al., in which an electrophoretic mobility shift assay was used to show that purified human Top1 binds to preformed intermolecular and intramolecular G4 structures [37]. Interestingly, this same study uncovered that Top1 promotes the formation of intermolecular G4 structures. When oligonucleotides (oligos) containing stretches of five or six consecutive guanines were incubated with purified human Top1, a protease-resistant four-stranded intermolecular complex of slower mobility was formed. This G4-formation activity was specific to Top1 and not observed with Top2, histone H2A, or BSA. Another set of experiments using human Top1 further confirmed that Top1 binds to G4 DNA-forming oligos as well as G-rich DNA and RNA oligos [38]. This study also showed that the cleavage of duplex DNA by human Top1 is inhibited by the presence of intermolecular or intramolecular G4 structures, and that this inhibition is due to the binding of Top1 to G4 DNA. It was also shown that duplex DNA cleavage by Top1 is inhibited by single-stranded, non-G4 capable DNA and RNA oligos containing stretches of two or three consecutive guanines. Pre-formed G4 oligos or guanine-rich single-stranded oligos were not cleaved by Top1. More recently, an effort to find oligonucleotide aptamer inhibitors of human Top1 confirmed that a variety of DNA oligos forming G quadruplex or quadruplex-duplex hybrid bind Top1 and compete it away from its substrate, dsDNA [39].

Many proteins have so far been identified as G4 DNA binding proteins. The protein-G4 DNA interactions can roughly be separated into three categories according to their effect on the stability of G4 structure; (1) promoting G4 DNA formation (yeast Rap1 and human thrombin) (2) stabilizing G4 DNA (murine nucleolin and human Ku protein) and (3) G4 DNA destabilizing (RecQ helicases BLM, WRN, and Sgs1) (Reviewed in [40]). More recently, a transcription factor Sub1 was identified as a potent G4 DNA binding protein that indirectly leads to destabilization of G4 via recruitment of the helicase Pif1 [41,42]. Sub1 or its mammalian homolog PC4, previously characterized as a single-strand DNA binding protein, binds to G4 DNA with a low Kd of ~2 nM but does not promote or disrupt G4 DNA folding [43]. Wildtype Top1, based on the experiments reported in Arimondo et al. can be considered to be in the category of proteins promoting G4 DNA formation [37,40]. In the future, it will be interesting to uncover how Top1 binds to G4 DNA structures. According to the X-ray crystal structure published, Top1 forms a tight, bi-lobed clamp around duplex DNA upon binding [14]. Since there is a significant difference between the diameters of duplex DNA and G4 DNA, which are approximately 2 nm and 2.4–2.8 nm, respectively [44,45,46], the Top1–G4 DNA complex possibly adopts a very distinct conformation from Top1-dsDNA. 

## 5. Functional Consequence of Top1–G4 DNA Binding

### 5.1. G4 Oligos as Top1 Inhibiting Aptamers

One possible functional consequence of the specific, high-affinity Top1–G4 interaction is the application of G4-forming oligos or aptamers as inhibitors of Top1. Aptamers are small single-stranded DNA or RNA oligos that are selected for a high affinity interaction with a specific target of interest (reviewed in [47]). Aptamers targeting proteins or other biologically relevant molecules have been selected by screening libraries of DNA or RNA oligos and further studied for the potential therapeutic application. Since it was first discovered that Top1 binds and is inhibited by G4-forming oligos [37,38], other similar results have been reported [39,48,49]. First, using purified Calf Thymus Top1, Shuai et al. characterized fourteen different guanine-rich oligos capable of forming either G-quadruplex or quadruplex-duplex hybrid and showed that all of the oligos act as competitive inhibitors of Top1 catalysis [39]. The inhibitory effect of these G4 aptamers were significantly more potent when they were treated with heat-cooling in presence of KCl to fold into G4 conformation. The IC_50_ of the G4-formed aptamers ranged from 0.1 to 2.7 µM. Top1 activity can be inhibited by small molecules such as camptothecin (CPT) that stabilize the Top1-cleavage complex (Top1-cc) and inhibit the ligation of the nicked substrate [50,51]. The mechanism of Top1 inhibition by the G4 aptamers, however, appeared to be mediated by the inhibition of the Top1-substrate binding. When Top1 was incubated with a mixture of a G4-forming aptamer and dsDNA substrate (i.e., pBR322 plasmid DNA), Top1 preferentially bound to the G4 aptamer [39]. 

The inhibitory effect of G4-forming oligos on Top1 activity was further confirmed in a study of guanine-rich microsatellite repeat sequences found in the human genome [48]. In this work, highly thermostable G4-forming oligos d(GGT)_4_ and d(GGGT)_4_ were added to Hela cell extract to determine how each of these oligos affects the Top1-mediated relaxation of supercoiled pUC19 plasmid DNA. Compared to a random oligo of similar length, which showed no effect in the relaxation assay, both G4 oligos d(GGT)_4_ and d(GGGT)_4_ were very effective in inhibiting Top1 with the IC_50_s of 0.63 and 0.12 µM, respectively. Another G-rich oligo d(GT)_16_, which is not capable of forming a thermostable G4 structure, did not inhibit Top1 activity, indicating the structure-specific nature of inhibition. When non-G4 flanking sequences were added to the effective inhibitor d(GGGT)_4_, the thermostability was decreased with the resulting T_m_ of 85 and 73 °C, respectively, for d(GGGT)_4_ and d(CACTGG-CC-(GGGT)_4_-TA-CCAGTG). But the longer oligo proved to be a more effective Top1 inhibitor with IC_50_ of 0.08 µM. In another study, six different guanine-rich aptamers were tested for their inhibitory effect on human Top1 [49]. These aptamers were previously designed to target other oncogenic proteins, namely STAT3, SP1, VEGF, NCL, and SHP-2, and commonly formed either parallel or anti-parallel G4 DNAs that are significantly stabilized by the presence of potassium cation as expected. All of these aptamers were very effective in inhibiting the activity of Top1 in a plasmid-relaxation assay with the IC_50_ in the low µM range. They were even more effective in inhibiting Top1 when compared to the aptamer first identified as a Top1-specific inhibitor by Shuai et al. [39]. The inhibition of Top1 by the G4 oligos was correlated with the inhibition of DNA replication, and this antiproliferative effect was specific to cancer cells [49]. Although the physical interaction between the G4-capable aptamers and Top1 was not determined in these studies, the specific nature of the inhibition strongly suggests that the competitive binding of the aptamers to Top1 underlies the inhibition of the catalytic activity. 

### 5.2. The Biological Consequence of the Top1 Interaction with G4 DNA

While the Top1-binding to G4 DNA is an interesting property to exploit in the use of G4-forming aptamers to target Top1 as a therapy, whether Top1 binding to endogenous, genomic G4 structures serves a biological function is not yet clear. Chromatin immunoprecipitation experiments conducted in yeast revealed that Top1 is enriched at telomeres, which contain G4 DNA forming sequences [52]. This study also demonstrated that the expression of the yeast Top1 catalytic mutant, Top1Y727F, in a *top1Δ* background elevates H4 K16 histone acetylation at genomic regions located proximal to telomeres. These results suggest that Top1 regulates transcription of telomere proximal genes and that the catalytic activity of Top1 is required for this function. It is possible that Top1 regulates chromatin state and expression of genes near telomeres through G4 DNA binding. Another possible function of the Top1–G4 DNA interaction is in the recruitment of G4-resolvases to the genomic G4 structures. Human Top1 was shown to interact with the Werner helicase, which can unfold G4 structures [53,54], suggesting that it is possible that Top1 promotes the localization of the Werner helicase to G4 structures through its own interaction with G4 DNAs. Top1 also interacts with the SV40 T antigen, which harbors DNA helicase activity [55]. These examples of Top1 interaction with the Werner helicase and the SV40 T antigen suggest further studies should be conducted to determine whether Top1 interacts with additional DNA helicases, particularly those helicases capable of unwinding G4 DNAs. 

### 5.3. Interaction between Mutant Top1 and G4 DNA In Vivo

**Figure 1:**
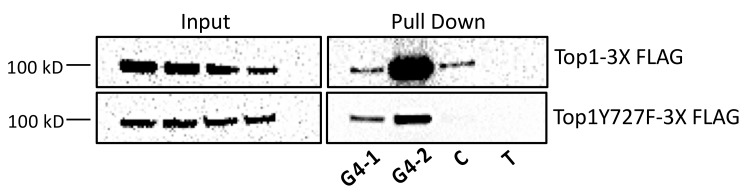
Yeast WT Top1 and Top1Y727F bind to G4 structures. Western blots of pulldowns of WT Top1-3XFLAG (top) and Top1Y727F-3XFLAG (bottom) from yeast whole cell lysates with biotinylated DNA oligonucleotides (MilliporeSigma). Biotinylated oligonucleotides G4-1, G4-2, C, and T were conjugated to Streptavidin-Coupled M-280 Dynabeads. Following the mechanical lysis of yeast cells with Biospec Mini-bead-beater, the cell lysate was collected and sonicated. Oligo-conjugated Dynabeads were incubated at 4 °C overnight with the yeast extract, washed, and then eluted by boiling in 1XSDS-PAGE loading buffer followed by immunoblotting analysis using anti-FLAG antibody to detect 3XFlag-tagged Top1 or Top1Y727F.

Even though the interaction of G4 DNA with the functional Top1 may result in transcriptional regulation or G4 structure resolution, other data suggest that the interaction of G4 DNA with Top1 catalytic mutant is deleterious. Human and *Saccharomyces cerevisiae* Top1 use amino acid residues tyrosine 723 and tyrosine 727, respectively, to undergo the nucleophilic attack of the phosphodiester DNA backbone effectively nicking the DNA [14]. However, if either of these residues is mutated to a phenylalanine, Top1 can bind, but not nick DNA. Interestingly, expression of Top1Y727F in yeast results in exacerbated recombination at a model G4-motif [34]. This elevated G4-induced recombination observed in the presence of Top1Y727F is significantly greater than the G4-induced recombination observed in a *top1Δ* yeast strain and is dependent on transcription. The effect of Top1Y727F on G4-induced genomic instability is surprising as the level of superhelical tension accumulation is expected to be similar in a *top1Δ* strain and a Top1Y727F-expressing yeast strain. Therefore, the increase in G4-induced genomic instability observed in a Top1Y727F-expressing yeast strain compared to a *top1Δ* strain must be from another factor in addition to negative supercoil accumulation. Yeast Top1Y727F was shown to be enriched at telomeres in chromatin immunoprecipitation experiments [52] and, in vitro, it preferentially binds to G4 oligos over a C-rich or a random control oligo (Berroyer and Kim, unpublished results, Figure 1, Table 1). Top1Y727F binding and stabilizing G4 structures would explain the highly elevated genomic instability at G4-motifs. Further, while WT Top1 may bind to G4 structures transiently, the lack of catalytic activity after DNA binding by yeast Top1Y727F may result in the trapping of Top1Y727F on G4 structures.

Another way Top1Y727F could increase the instability of G4 DNA-forming genomic loci is by binding to G4 DNA and then preventing G4 resolvases from accessing the structure. This mechanism has been described for the human protein nucleolin (NCL) [56]. NCL is an essential nuclear/nucleolar protein with multiple known functions [57]. Most prominently, NCL is involved in the ribosomal RNA maturation. Mammalian NCL, however, was also shown in vitro to be capable of high-affinity binding to G4 DNA with Kd in the low nM range [58,59]. More recently, the specific interaction between NCL and the G4 DNA formed from the (GGGGCC)n repeat in the human C9orf72 gene was demonstrated in vivo [60]. Expansion of the (GGGGCC)n repeats in the C9orf72 gene is associated with the neurological disorders amyotrophic lateral sclerosis (ALS) and frontotemporal dementia (FTD). An in vitro helicase assay has shown that G4 DNA, when in complex with NCL, becomes resistant to unwinding by Werner helicase, a G4 resolvase [56]. If G4 structures are left unresolved, they could be potent blocks to DNA replication leading to genome rearrangements [61,62,63]. Similarly, it has been shown that Nsr1, the yeast homolog of NCL, binds to G4 structures in vitro and in vivo [64]. The deletion of *NSR1* gene or the expression of a truncated form of Nsr1 missing an important G4 DNA-binding domain in a *nsr1Δ* background significantly reduces recombination at a model G4-motif. This indicates that Nsr1, like NCL, increases G4-induced instability through G4 binding. Of note, *NSR1*-deletion also reduces G4-induced recombination in a *TOP1Y727F* background, however, not to wild type levels as observed in *top1Δ nsr1Δ* strain (Berroyer and Kim, unpublished results). Because Nsr1 and Top1 physically interact [65], it is possible that these two G4 DNA binding proteins form a higher-order complex to increase G4-induced instability in a synergistic manner. 

### 5.4. DNA–Protein Complexes as DNA Replication Barriers

Replication forks can stall and collapse at DNA lesions leading to genome instability. Such genotoxic impediment to replication can include DNA–protein complexes (reviewed in [66]). Especially, proteins covalently trapped on DNA are known to be natural replication blocks. During the removal of helical tension, Top1 forms a phospho-tyrosyl bond with the 3′ end of nicked DNA, and this covalent DNA–protein complex is termed a Top1-cleavage complex (Top1-cc) [14]. After strand swiveling has occurred, the 5′ OH of the nicked strand attacks the phosphor-tyrosyl bond connecting Top1 to DNA which results in the ligation of DNA and release of the enzyme. Top1-ccs, such as those induced by the Top1 poison camptothecin (CPT) and its derivatives, block DNA replication and induce DNA strand breaks and recombination (reviewed in [51]). On the other hand, there are examples of non-covalent proteins-DNA complexes that block DNA replication machinery in both prokaryotes and eukaryotes. In *Escherichia coli*, a protein called Tus binds to Ter sites within DNA, and this DNA–protein interaction terminates replication in a unidirectional manner to prevent the collision of replication forks approaching each other in a head-on orientation [67,68,69]. The replication fork barrier created by the Tus-Ter interaction functions to ensure proper replication termination of the circular chromosome [70]. Another example of non-covalent DNA–protein complex that can block replication in *E. coli* is the array of lacI repressor molecules bound to Lac operon [71]. In eukaryotes, tight DNA–protein interactions that inhibit DNA replication occur at the highly transcribed genomic loci that encode ribosomal RNA. In *S. cerevisiae*, similar polar Replication Fork Blocks (RFBs) are generated at each of the 150–200 rDNA tandem repeats located at Chromosome XII [72]. Fob1 binding at the rDNA array on chromosome XII is an example of the DNA–protein complex serving as replication fork block [73]. In yeast cells defective for the DNA helicase Rrm3, replication was blocked at discrete genomic loci including known heterochromatin regions such as centromeres and silent mating type loci but also at tRNA genes, inactive replication origins, and transcriptional silencers [74]. These regions are characterized by the non-histone DNA–protein complexes, indicating that high-affinity DNA–protein complexes can form replication blocks or barriers. 

DNA secondary structures such as G4 DNA also form potent replication barriers (reviewed in [66]). G4 DNA in the template strand stalls the replicative polymerases in vitro and translesion DNA polymerases such as the mammalian Pol eta and kappa and bacterial Pol IV are necessary for continued synthesis [62,75,76,77,78]. Replication in yeast is impeded at G4 motifs when the G4 DNA helicase Pif1 is disrupted [63,79,80]. G4 DNA stabilized by small molecule ligands such as pyridostatin (PDS) or PhenDC3 increase the instability at G4-forming sequences in the eukaryotic genomes indicating that the stability of G4 DNA correlates with its efficacy as replication block [81,82,83,84]. Similarly, a more problematic obstacle to replication is expected when G4 DNA is in complex with high-affinity binding protein such as the Top1 catalytic mutant (Top1Y727F), which binds to G4 DNA to form a stable complex (Figure 1, Table 1). When the Top1Y727F mutant was expressed in *top1Δ* yeast cells, there was a significant and G4-specific elevation in the recombination rate [34]. On the other hand, expression of the Top1T722A mutant, which can competently cleave DNA but is defective in the re-ligation step leading to the formation of Top1-cc, resulted in a modest elevation in recombination independent of the sequence. CPT-treatment also led to a non-specific elevation of recombination. Together, these data indicate that the G4-specific elevation of recombination upon expressing Top1Y727F is not due to inhibition of Top1 catalytic activity or due to increased formation of Top1-cc. The preferential binding of the mutant protein to G4 DNA is then one mechanism possibly underlying the sharp increase in G4-specific recombination following the expression of Top1Y727F. That is, Top1 mutants bound tightly to G4 DNA structures could be strongly disruptive to replication and recombinogenic in a manner similar to those covalent and non-covalent DNA–protein complexes that function as replication barrier sites. 

While proteins that are covalently trapped or are tightly bound to DNA are known to block replication, they can be resolved to prevent genomic instability in several different manners. In yeast, the protease named weak suppressor of *SMT3* protein 1 or Wss1, degrades proteins trapped on DNA, including Top1-ccs [85]. Top1-ccs become SUMOylated in yeast, and the SUMOylation of proteins trapped on DNA by DNA-bound SUMO ligases directs and enhances the recruitment of Wss1, which contains SUMO-interacting motifs. Wss1 degrades DNA-trapped proteins almost entirely, leaving behind small peptide remnants that are further processed by the proteasome or are bypassed during replication by translesion polymerases. The human homolog of Wss1 is a protein named Spartan; the loss of Spartan leads to an accumulation of unrepaired Top1-ccs in human cells [86,87,88]. However, instead of SUMO-interacting motifs as in yeast Wss1, the recruitment and activity of Spartan is regulated by its ubiquitin-binding domain [89]. In the future, it will be interesting to determine if Top1 mutants trapped on G4 DNAs are modified by SUMOylation and/or ubiquitylation and subsequently processed by Wss1 and Spartan in *S. cerevisiae* and humans, respectively.

## 6. Top1 Mutants in Cancer Cells and G4-Induced Genomic Instability

Top1 has long been the molecular target of chemotherapeutics. CPT and CPT derivatives are widely used to treat many cancers, and work by stabilizing Top1-ccs as mentioned above. Cancer cells can become resistant to CPT through multiple different mechanisms (reviewed in [90]). First, consistent with the cell culture experiments demonstrating that the reduced levels of Top1 confer resistance to CPT, cancers relapsing after chemotherapy spontaneously become resistant to CPT through reduced expression of Top1 [91,92]. Cancer cells can also become impervious to CPT-treatment through mutations of Top1 that reduce the ability either to bind to duplex DNA or to cleave duplex DNA following binding. A study conducted in yeast found intragenic suppressors of the yeast Top1T722A mutation [93], which mimics the cytotoxic activity of CPT. One such suppressor mutant, yeast Top1G369D, has a critically reduced duplex DNA binding ability. Another study found that T729 is mutated in a human lung cancer cell line that is resistant to the CPT analog irinotecan [94]. Further investigation demonstrated that expression of the human Top1T729K and Top1T729E mutants in yeast confers resistance to CPT, and that these Top1 mutants are defective in duplex DNA binding [95]. Other studies have demonstrated that Top1 catalytic mutants, which harbor reduced duplex DNA cleavage abilities, exist in cancer cell lines and cancer patients. The homozygous Top1G365S mutant, which was found in a colon cancer cell line that is resistant to the active metabolite of irinotecan, displays 50% reduced catalytic activity [96]. Another mutant, Top1W736Stop, was found in a non-small cell lung cancer patient treated with irinotecan and is predicted to have reduced catalytic activity [97]. A large number of Top1 mutations identified in CPT-resistant cancer cells and patient samples remain uncharacterized. Some of the mutations located in the catalytic C-terminal domain of human Top1 (amino acids 713–765) are listed in Table 2. Although these are missense or nonsense mutations located in the catalytically critical domain of the protein, the residues in the C-terminal domain also participate in the contact with dsDNA [13,14]. For a majority of those listed mutations, whether each of the mutations results in the loss of DNA cleavage or DNA binding activity of Top1 has not been studied. Of note, additional mutations not listed that are located in the core domain of Top1 can also result in reduced enzyme catalysis, like the G365S mutation found in a CPT-resistant colorectal cancer cell line as described above [96].

Top1 is required to suppress recombination and instability at a highly transcribed G4-motif in yeast and suppresses the genotoxic effect of G4-ligands in human cells [33,34,35]. siRNA-mediated knock-down of Top1 in mouse cells elevates CSR and chromosomal translocations involving the G4 DNA-forming IgH switch region sequences [31,32]. The reduced levels of Top1 in the CPT-resistant human cancer cells is then expected to disrupt its normal cellular role of preventing the excess level of negative supercoiling at highly transcribed loci and to thereby elevate the accumulation of non-B DNA formation including G4 DNA. The reduced Top1 level and also the reduced Top1 function in cells with DNA-binding defective mutants (e.g., Top1T729K and Top1T729E) very likely manifest in the elevated genome instability associated with the formation of G4 DNA and other non-B structures. However, in those CPT-resistant cancers with Top1 mutants that are catalytically defective but competent for DNA binding, additional consideration must be made according to some interesting preliminary data from yeast studies. The expression of yeast Top1 mutant Y727F further exacerbates the instability at G4 DNA-forming genomic loci beyond the loss of Top1 function by additionally binding to G4 DNA and other G4 DNA-binding proteins [64]. It needs to be examined whether a similar G4-specific deleterious effect is produced with the expression of human Top1 mutants such as Top1G365S and Top1W736Stop mutants, which can bind but not cleave DNA. Incidences of secondary cancers following treatment with the CPT-derivative irinotecan have been documented. In one case, a patient with X-linked agammaglobulinemia and metastatic colorectal cancer experienced an increase in cancer progression and severe hypocalcemia after treatment with irinotecan [103]. Another study revealed that a colon cancer patient developed secondary acute promyelocytic leukemia following treatment with irinotecan and oxaliplatin [104]. While both Top1 mutations and secondary genomic rearrangements have been discovered in cancer patients treated with CPT or CPT derivatives, a link between these documented Top1 mutations and G4-induced secondary genomic rearrangements has not been studied. In the future, it will be important to uncover if cancer cells from patients treated with CPT or CPT derivatives have increased secondary genomic rearrangements at G4-forming loci. The increased potential for the G4-induced genome instability due to the catalytic Top1 mutant could render cancers more complicated to treat and potentially worsen patient outcomes.

## 7. Concluding Remarks

**Figure 2:**
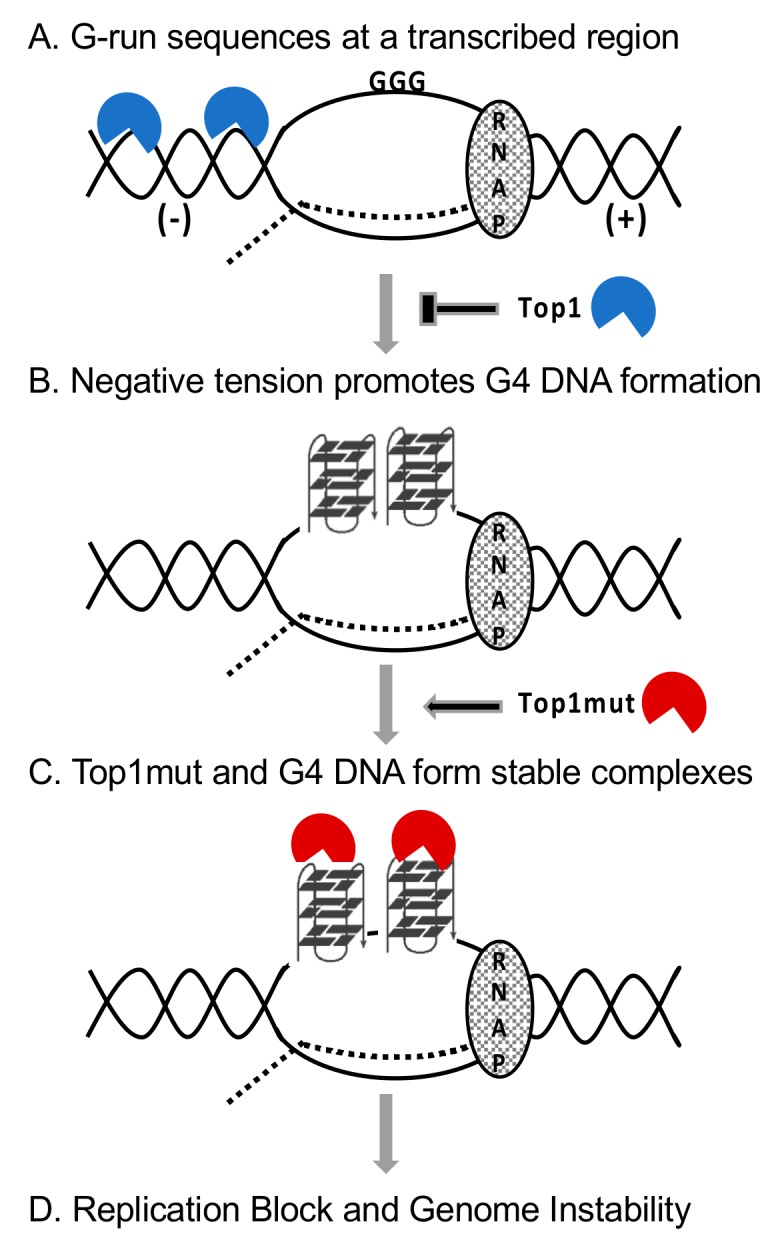
A model of genome instability induced by co-transcriptionally formed G4 DNA and the effect of Top1 activity and mutation. RNAP—RNA polymerase complex. Dotted line—the nascent transcript. (−)—negative tension behind the transcription complex. (+)—positive tension ahead of the transcription complex. Top1mut—Top1 mutant.

Given the significance of G4 DNA-forming sequences in the cancer-associated genome rearrangements, it is very important to study how the guanine-run containing sequences are converted into hotspots of genome instability. As shown in yeast and mammalian systems, Top1 plays an essential role in preventing genome instability at these G4 DNA-forming loci by removing transcription-associated superhelical tension and maintaining the proper topological conformation (Figure 2A,B). Furthermore, newly emerging evidence indicate that Top1 can specifically and preferentially interact with G4 DNA. The interaction between Top1 mutants and G4 DNA, however, could lead to the formation of pathological complexes that interfere with efficient DNA replication (Figure 2C,D). Based on the preliminary data in yeast model system linking the expression of certain Top1 mutants with the acutely elevated G4 DNA-associated genome instability, further characterization of human Top1 mutants, particularly those arising in CPT-treated cancer cells, could shed light on the significance of the interaction between mutant proteins and the non-B DNA structure. On the other hand, the significance of the Top1–G4 DNA interaction can be viewed from a very different perspective. Multiple recent studies suggest that G4 DNA-forming oligos can preferentially bind and interfere with the activity of Top1, which has been a very important target of anti-cancer therapy. This property can be exploited to develop a new class of drugs targeting Top1 that can serve as an alternative and a complement to the Top1-poisoning CPT and CPT derivates. 

## Figures and Tables

**Table 1:** The sequences of the oligonucleotides used in pull down assay. Guanine runs are underlined and italicized.

Oligonucleotide	Sequence
G4-1	5’ GAGCT*GGGG*TGAGCT*GGG*CTGAGCT*GGGG*TGAGCT*GGG*CTGAGCT
G4-2	5’ A*GGG*CTCTGCCTT*GGGGGGGGGG*CAGGAA*GGG*A
C	5’ AGCTCAGCCCAGCTCACCCCAGCTCAGCCCAGCTCACCCCAGCTC
T	5’ GCACGCGTATCTTTTTGGCGCAGGTG

**Table 2:** A selected list of human Top1 C-terminal mutations from studies, cancer cell lines, and patient samples.

Top1 Mutant	Origin	Reference and Mutation ID
Q713H	breast cancer tissue tumor sample	ICGC(BRCA-US); COSMIC Genomic Mutation ID COSV63696584
I714T	human colorectal cancer cell line	[98]; COSMIC Genomic Mutation ID COSV63693067
R727S	urinary tract cancer tumor sample	[99]; COSMIC Genomic Mutation ID COSV99057653
T729A	irinotecan-resistant human lung cancer cell line	[94]
W736C	urinary tract cancer tumor sample	ICGC/GDC(BLCA-US); COSMIC Genomic Mutation ID COSV63695286
W736STOP	non-small cell lung cancer patient treated with irinotecan	[97]
E741STOP	urinary tract cancer tumor sample	[100]; COSMIC Genomic Mutation ID COSV63696859
T747P	prostate cancer tumor sample	[101]; COSMIC Genomic Mutation ID COSV63695236
R749W	soft tissue/smooth muscle tumor sample	[100]; COSMIC Genomic Mutation ID COSV63696078
R749Q	large intestine carcinoma sample	ICGC(COAD-US); COSMIC Genomic Mutation ID COSV63696579
A753S	liver cancer tumor sample	ICGC(LICA-CN); COSMIC Genomic Mutation ID COSV63692735
A759T	large intestine carcinoma tumor sample	ICGC(COCA-CN); COSMIC Genomic Mutation ID COSV63694655

ICGC, International Cancer Genome Consortium (https://icgc.org); COSMIC, Catalogue of Somatic Mutations in Cancer (see reference Tate et al., 2018 [102] and cancer.sanger.ac.uk).
